# Targeted Prostatic Delivery of Levofloxacin via a Novel Vas-Deferens Injection Device: A Pharmacokinetic Study in Rats

**DOI:** 10.3390/pharmaceutics18080931

**Published:** 2026-07-29

**Authors:** Haiming Cao, Junjie Wu, Mingwei Zhan, Bo Ma, Qi Zhang, Xuejun Shang

**Affiliations:** 1 The Department of Urology, Nanjing Jinling Hospital, Affiliated Hospital of Medical School, Nanjing University, Nanjing 210007, China; chmmaths@foxmail.com (H.C.);; 2 The Department of Urology, Hangzhou TCM Hospital of Zhejiang Chinese Medical University, Hangzhou 310007, China; 3 The School of Pharmacy, Nanjing Technology University, Nanjing 211816, China; 4 State Key Laboratory of Pharmaceutical Biotechnology, Nanjing University, Nanjing 210023, China

**Keywords:** vas deferens, levofloxacin, prostate, targeted drug delivery, pharmacokinetics

## Abstract

**Objective:** Current pharmacological treatments for prostate diseases are limited by poor drug penetration into the prostate and systemic adverse effects. This study evaluated whether a novel vas-deferens injection device could improve targeted delivery of levofloxacin to the prostate. **Methods:** Healthy adult male Sprague–Dawley rats received a single dose of levofloxacin by either intravenous administration (IV) or trans-vas-deferens administration using a novel disposable device (VS). Plasma and prostate samples were collected from 0.5 to 24 h and analyzed by LC-MS/MS. Iodixanol micro-CT imaging was used to visualize the local delivery pathway. Pharmacokinetic evaluation included observed composite-profile Cmax/Tmax, AUC0-24, and fT. Time-dependent tissue selectivity was evaluated through partial AUC analyses, prostate-to-plasma exposure ratios, and pointwise concentration ratios. A bioequivalence-style framework was applied to compare relative exposure parameters using geometric mean ratios (GMRs) with the 80.00–125.00% reference interval. Exploratory PK/PD evaluation combined literature-derived free-drug fractions (fut_plasma = 0.55, fut_prostate = 0.080) to estimate fAUC/MIC against representative uropathogens, supplemented by Monte Carlo simulation (*n* = 5000) to estimate probability of target attainment (PTA) at an fAUC/MIC ≥ 30 threshold. **Results:** Imaging confirmed selective distribution within the reproductive tract after vas-deferens delivery. In plasma, VS substantially reduced systemic exposure compared with IV: Cmax was 4.74 μg/mL (VS) versus 10.72 μg/mL (IV), and AUC0-24 was 15.47 versus 28.59 μg·h/mL. In the prostate, VS maintained comparable or numerically higher exposure: Cmax was 61.96 μg/g (VS) versus 52.02 μg/g (IV), and AUC0-24 was 235.77 versus 208.77 μg·h/g. The tissue distribution factor fT was substantially elevated in VS (2.26; 95% CI: 1.58–3.19) compared with IV (1.06; 95% CI: 0.89–1.30). Bioequivalence-style analysis demonstrated that plasma GMRs for AUC0-24 and Cmax fell well below 80%, confirming reduced systemic burden, while prostate GMRs were maintained, and the prostate-to-plasma AUC ratio GMR was increased. Time-dependent partial AUC analyses revealed that VS significantly elevated the prostate-to-plasma exposure ratio. PK/PD evaluation showed that at 24 h, prostate fAUC/MIC against Enterobacteriaceae (MIC = 0.5 μg/mL) was 37.7 (VS) versus 33.4 (IV), with corresponding PTA values of 97% and 94%, while plasma fAUC/MIC remained substantially lower in the VS group. **Conclusions:** Trans-vas-deferens administration of levofloxacin via a novel injection device effectively reduced systemic drug exposure while maintaining or enhancing prostate tissue concentrations, thereby widening the therapeutic window and reducing systemic drug exposure.

## 1. Introduction

The National Institutes of Health (NIH) classifies prostatitis into four categories, with chronic bacterial prostatitis (CBP) designated as Type II. CBP accounts for 7–14% of all prostatic diseases in Europe [1,2]. As a common inflammatory condition of the male urinary system predominantly affecting men under 50 years old, CBP is clinically characterized by recurrent urethral irritation symptoms, urethral discharge, chronic pelvic pain, and sexual dysfunction. Although non-life-threatening, the condition imposes significant economic and psychological burdens on both patients and society [3,4]. The vast majority of bacterial prostatitis cases are caused by Escherichia coli [5].

The unique anatomical structure and physiological barriers of the prostate, combined with frequent polymicrobial infections, present significant challenges in treating prostatic diseases. The prostatic lipid envelope and blood-prostate barrier (BPB) substantially impede effective diffusion and accumulation of most antibiotics from systemic circulation into prostatic tissue, thereby compromising therapeutic efficacy [6,7]. Although conventional therapeutic approaches including oral administration, intravenous injection, and local injections can provide symptomatic relief to some extent, they demonstrate limited efficacy in improving cure rates and reducing recurrence, while frequently contributing to drug resistance development. Systemically administered drugs undergo extensive distribution before reaching the prostate, often failing to achieve effective tissue concentrations while potentially causing systemic adverse effects. Local injections face clinical limitations due to procedural complexity, heterogeneous drug distribution, and poor patient compliance. Although physical therapies may provide short-term symptomatic relief, they generally fail to address fundamental inflammatory or hyperplastic pathologies [8,9].

In the treatment of chronic bacterial prostatitis (CBP), antimicrobial agents serve as primary therapeutic interventions. However, the prostate’s specialized vascular supply and the BPB restrict penetration of many drugs. Similar to the blood–brain barrier, the BPB limits transmission of water-soluble drugs while permitting partial penetration of lipid-soluble agents [10,11]. Nevertheless, studies demonstrate that antibiotics administered orally or intravenously typically achieve only subtherapeutic concentrations in prostatic tissue, substantially complicating CBP treatment and leading to frequent disease recurrence and antimicrobial resistance development [12].

As a broad-spectrum antimicrobial agent, levofloxacin is widely employed in treating genitourinary tract infections including prostatitis, owing to its favorable tissue penetration and antibacterial efficacy [13,14]. Although its lipophilic properties enable partial penetration through the blood-prostate barrier (BPB), systemic administration fails to achieve therapeutic concentrations sufficient for optimal antibacterial activity within prostatic tissue [15]. Consequently, enhancing localized levofloxacin concentrations in the prostate emerges as a crucial determinant for treatment optimization.

Current strategies for enhancing therapeutic efficacy in prostate diseases primarily encompass three approaches: modification of administration routes (e.g., intraprostatic injection and transrectal delivery) [16,17], physical enhancement of drug permeability (including photodynamic/thermal therapy and ultrasound-mediated methods) [18,19], and advanced drug delivery systems (such as liposomal carriers, prostate-targeting agents, and blood-prostate barrier modulation) [20,21,22]. Among these, route modification represents the most readily implementable strategy. This consideration prompted the development of a novel vas deferens injection device for prostatic drug delivery.

Trans-vas-deferens injection emerges as an innovative minimally invasive localized approach that may offer distinct therapeutic advantages. This technique enables direct drug administration to the prostate via interventional access, facilitating immediate and targeted tissue exposure. The localized delivery paradigm not only achieves substantially elevated intraprostatic drug concentrations but simultaneously reduces systemic drug distribution, thereby minimizing potential adverse effects. Furthermore, the vas deferens puncture technique demonstrates operational simplicity with minimal tissue trauma, showing promising drug delivery efficiency in clinical evaluations.

This study aims to compare the effects of intravenous administration versus trans-vas-deferens injection therapy on levofloxacin concentrations in the rat prostate, and to evaluate the efficacy of a novel trans-vas-deferens injection device as a localized drug delivery approach. This study will provide theoretical support and technical references for localized treatment of prostatic diseases.

## 2. Materials and Methods

### 2.1. Animals

Specific-pathogen-free (SPF) Sprague–Dawley (SD) rats, sexually mature, 8 weeks old, and weighing 250–300 g, were supplied by GemPharmatech Co., Ltd. (Nanjing, Jiangsu, China). The animals were housed in an environmentally controlled room maintained at 23 ± 3 °C and 55 ± 8% relative humidity with a 12 h light/dark cycle. All experimental procedures were approved by the Institutional Animal Care and Use Committee (IACUC) of Affiliated Jinling Hospital, Medical School of Nanjing University (Ethical approval No. DZYJSKTB20240720001) and conducted in accordance with the National Institutes of Health Guide for the Care and Use of Laboratory Animals.

### 2.2. Injection Method by the Novel Disposable Vas-Deferens Injection Device

Male SD rats were anesthetized with 25% urethane. A 2 cm left-paratesticular skin incision was made, the peritoneum was opened, and the left testis, epididymis, and vas deferens were exteriorized under a surgical microscope (10× magnification).

**Figure 1:**
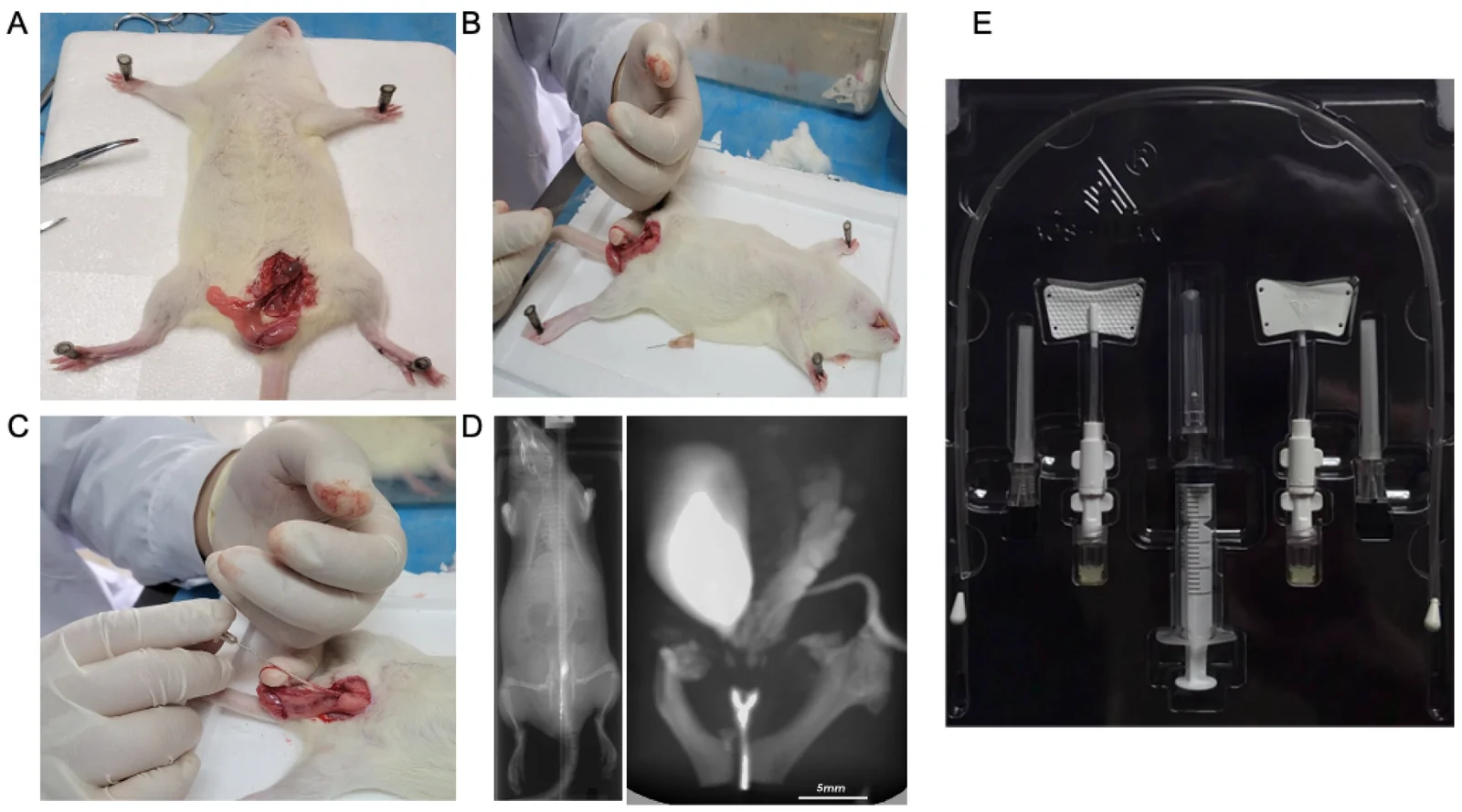
Novel disposable vas deferens injection device and validation of the method. (**A**–**C**) Schematic illustration of the vas-deferens injection procedure in rats: (**A**) positioning of the vas deferens, (**B**) insertion of the needle at 45°, and (**C**) slow drug infusion followed by compression of the puncture site. (**D**) Representative micro-computed tomography image of the rat reproductive system after injection of iodixanol contrast agent via the vas deferens device. (**E**) Photograph of the disposable vas deferens injection device, consisting of a 29-gauge puncture needle and a 0.2 mm micro-catheter.

The vas deferens was gently lifted with micro-forceps and stabilized on the operator’s finger. A novel disposable vas deferens injection device (Patent number: ZL20130211746.2 in China) consisting of a 29 G puncture needle and a 0.2 mm micro-catheter was inserted at a 45° angle. Loss-of-resistance and visual confirmation of the needle tip inside the vas lumen indicated successful puncture. The catheter was advanced 1–2 cm into the lumen, the needle was withdrawn, and the catheter was connected to a 1 mL syringe containing levofloxacin (45 mg kg^−1^ in 0.2 mL sterile saline). The drug was injected slowly (≤20 µL s^−1^); blanching and mild distension of the vas deferens confirmed delivery. After the catheter was removed, the puncture site was compressed for 30 s to prevent reflux (Figure 1A–C).

Wounds were sutured if survival exceeded 1 h, or covered with saline-moistened gauze for shorter intervals. For pharmacokinetic sampling, blood was collected immediately before tissue harvest and prostate tissue was obtained after euthanasia at 0.5, 1, 2, 4, 6, 8, 12, and 24 h post-dose. Because prostate sampling was terminal, different animals contributed to different scheduled terminal time points.

In both IV (intravenously) and VS (trans-vas-deferens) groups, blood was drawn from the central vein (posterior vena cava) at the scheduled sampling time point into heparinized tubes and centrifuged to obtain plasma (10 min, 2000–3000 rpm). Prostate samples were collected from the same terminal procedure, homogenized using 100 mg of tissue in 1 mL normal saline, snap-frozen in liquid nitrogen, and stored at −80 °C until LC-MS/MS analysis. The experiment was performed in three sequential stages, including injection, tissue collection, and analysis, and each stage was conducted by different personnel. To ensure blinding, only the personnel responsible for the injection procedure and the overall experimental coordination were aware of the group assignments.

### 2.3. Study Design

SPF-grade male Sprague–Dawley rats were randomly allocated to vas deferens intervention groups or intravenous groups (6–7 SD rats per group). Each animal received a single dose of levofloxacin (purity: 98%, lot no. C15134411, Shanghai Macklin Biochemical Technology Co., Ltd., Shanghai, China) at 45 mg kg^−1^ (the calculation is based on a daily dose of 500 mg for a 70 kg adult, to simulate the daily dose for a human). In the VS group, dosing was performed via the novel vas deferens device; in the IV group, dosing was performed by tail-vein injection. Animals were scheduled for sample collection at 0.5, 1, 2, 4, 6, 8, 12, and 24 h after administration. Plasma and prostate samples were then collected for LC-MS/MS analysis. At each scheduled terminal time point, the plasma and prostate samples were therefore paired within the same animal, allowing calculation of an individual prostate-to-plasma concentration ratio for that time point.

### 2.4. Determination of Levofloxacin Concentrations by LC-MS/MS

A 100-μL aliquot of rat plasma was spiked with 10 μL of internal standard (Levofloxacin-d8, 10 μg mL^−1^, purity: 99.84%, lot no.HY-B0330S, MedChemExpress, Monmouth Junction, NJ, USA), deproteinized with 300 μL of methanol, vortex-mixed, and centrifuged at 15,000× *g* for 10 min at 4 °C. One hundred microliters of the supernatant were transferred to an autosampler vial and injected into the LC-MS/MS system (PreMed 5200, EXPEC Technology Co., Ltd., Hangzhou, China). Levofloxacin concentrations in plasma and prostate were quantified using a fully validated high-performance liquid chromatography–tandem mass spectrometry method. Separate calibration curves were constructed for each matrix (Figure S1). Results are expressed as μg mL^−1^ for plasma and μg g^−1^ for prostate tissue.

Liquid-phase parameters: The chromatographic column was a Shim-pack GIST 3 μm C18-AQ column (3 × 100 mm, 3 μm). Mobile phase A was 0.1% formic acid in water, and mobile phase B was acetonitrile. The flow rate was 0.5 mL/min, the column temperature was 35 °C, and the injection volume was 5 μL. Gradient elution: 0–0.5 min: 90% (A); 0.5–1 min: 90–30% (A); 1–2.5 min: 30–2% (A); 2.5–3 min: 2% (A); 3–3.1 min: 2–90% (A); 3.1–4.2 min: 90% (A). MS parameters: Electrospray ionization (ESI), positive ion MRM scan mode, ion pairs 362.17 → 261.46 *m*/*z* (levofloxacin), 370.17 → 265.46 m/z (internal standard, levofloxacin-D8), collision energy 30 V for both. Atomisation gas flow rate: 1.8 L/min; desolvation gas temperature: 500 °C; desolvation gas flow rate: 12 L/min; backflush gas flow rate: 1.1 L/min; capillary high voltage: 4.6 kV; collision gas flow rate: 0.45 mL/min.

### 2.5. Reproductive-System Contrast Imaging via the Novel Disposable Vas Deferens Injection Device

Using the aforementioned vas deferens injection technique, iodixanol was administered into the rat vas deferens, and reproductive system images were acquired with a micro-CT scanner.

### 2.6. Noncompartmental Analysis of Levofloxacin via Vas Deferens and Intravenous Injection in Rats

Non-compartmental analysis (NCA) of the concentration–time profiles was performed with R version 4.3.3 using the PKNCA package (v 0.12.0). Pharmacokinetic parameters included the highest observed composite concentration (Cmax), the corresponding scheduled sampling time (Tmax), and the area under the concentration–time curve (AUC) calculated by the linear trapezoidal rule. Organ drug penetration was expressed as the ratio of the AUC in tissue (AUCorgan) to the AUC in plasma (AUCplasma). Non-parametric bootstrapping (*n* = 2000) was performed to obtain confidence intervals (CI) for the model parameters. For NCA input formatting, concentration at time zero (C_0_) was set to zero in both groups. For the IV group, the first observed concentration (0.5 h) was used as Cmax; no back-extrapolation to estimate a theoretical C_0_ was performed. NCA was performed on a single composite concentration-time profile per group (sparse terminal-sampling design). Because only one point estimate is obtained per parameter under this design, no direct SD can be calculated. Variability was estimated by nonparametric bootstrapping (2000 resamples) and is expressed as bootstrap mean [95% CI]. Tmax is reported as bootstrap median [95% CI].

To characterize the time dependence of exposure differences, AUC0-t values were calculated at each predefined window endpoint (0.5, 1, 2, 4, 6, 8, 12, and 24 h). Partial AUC is useful for distinguishing early systemic exposure differences from later tissue-retention phenomena and therefore provides more mechanistic information than a single AUC0-24 estimate.

At each time window, prostate and plasma partial AUC values derived from the same group-level composite profiles were used to calculate the prostate-to-plasma exposure ratio:

AUC ratio (0-t) = AUCprostate (0-t)/AUCplasma (0-t)

This ratio quantifies tissue selectivity relative to systemic exposure. Tissue-to-plasma AUC ratios are widely used in anti-infective pharmacology to evaluate whether drug exposure at the infection site is sufficient to support therapeutic activity.

In addition to cumulative exposure, matched pointwise concentration ratios were calculated:

Concentration ratio (t) = Cprostate (t)/Cplasma (t)

This ratio reflects instantaneous tissue distribution at each sampling time and complements the cumulative information provided by AUC-based metrics [15,23].

### 2.7. Free-Drug Concentrations Conversion and Tissue Distribution Factor

Because antibacterial activity is mainly driven by free drug exposure, total concentrations were converted to estimated free concentrations using prespecified free fractions:

Cfree, plasma = Ctotal, plasma × fu_plasma

Cfree, prostate = Ctotal, prostate × fut_prostate

In this study, fu_plasma was set to 0.55, based on the rat levofloxacin plasma unbound fraction reported in microdialysis-based population pharmacokinetic work. The prostate free fraction fut_prostate was set to 0.080, consistent with prior semimechanistic and translational levofloxacin prostate-distribution modeling frameworks that parameterized free drug distribution in the prostate relative to plasma. Because the present dataset consisted of total concentrations rather than directly measured free interstitial concentrations, these calculations should be interpreted as literature-informed estimates of free exposure.

The tissue distribution factor fT was defined using the 0–24 h exposure interval:

fT = AUC0-24, prostate, free/AUC0-24, plasma, free

Substituting total exposure and free fractions yields:

fT = (AUC0-24, prostate, total × fut_prostate)/(AUC0-24, plasma, total × fu_plasma)

This formulation is consistent with the logic used in studies comparing target-tissue free exposure with systemic free exposure to quantify tissue penetration and retention [24,25].

### 2.8. Bioequivalence-Style Relative Exposure Analysis

A bioequivalence-style statistical framework was used to standardize the comparison of relative exposure between regimens. This framework is particularly suitable for exposure-related pharmacokinetic metrics such as AUC and Cmax because it simultaneously provides effect direction, effect size, and interval uncertainty.

For the plasma analysis, AUC0-24 and Cmax were analyzed. For the prostate analysis, AUC0-24, AUC0-24 prostate/AUC0-24 plasma, and Cmax were analyzed. All parameters were evaluated on the natural-log scale and converted back to the original scale as geometric mean ratios (GMRs):

GMR = exp[mean(ln(Test)) − mean(ln(Reference))]

When expressed as percentages:

GMR (%) = 100 × exp[mean(ln(Test)) − mean(ln(Reference))]

The conventional 80.00% to 125.00% reference range was used as an interpretive interval. In the present study, this interval was not used for regulatory equivalence claims, but rather as a standardized benchmark to judge whether systemic exposure decreased and whether local prostate exposure was preserved or enhanced. Tmax was summarized descriptively because it is not usually handled as a primary log-transformed interval endpoint in standard bioequivalence guidelines [26,27].

### 2.9. Exploratory PK/PD (Pharmacokinetics/Pharmacodynamics) Analysis Based on fAUC/MIC

For fluoroquinolones, antibacterial activity is primarily exposure-driven, and fAUC/MIC is one of the principal PK/PD indices:

fAUC/MIC = fAUC/MIC

where fAUC is the free-drug area under the concentration-time curve and MIC is the minimum inhibitory concentration. Because only unbound drug is assumed to distribute and interact with bacterial targets, PK/PD analyses generally use free exposure rather than total exposure [28,29,30].

The MIC is the lowest antimicrobial concentration that prevents visible bacterial growth under standardized in vitro conditions and is a central quantitative endpoint in antimicrobial susceptibility testing. In the present analysis, representative pathogens and their corresponding representative MIC values were prespecified for scenario-based comparison. The pharmacodynamic target threshold was set as fAUC/MIC ≥ 30. This threshold was used as a practical comparative benchmark within the present dataset and is consistent with the use of fAUC/MIC as a central fluoroquinolone PK/PD target framework. Because optimal targets may vary according to pathogen, infection site, and outcome definition, this threshold should be interpreted as a reference target for comparative evaluation rather than a universal breakpoint.

### 2.10. Monte Carlo Simulation and PTA

Monte Carlo simulation was performed to extend the sample-based fAUC/MIC findings to a larger virtual population and to estimate the probability of target attainment (PTA). The simulation assumed that fAUC values at the selected windows followed an approximately log-normal distribution after natural-log transformation, a standard working assumption for positively skewed exposure variables in pharmacokinetics [31].

For each selected time window, the mean (mu_log) and standard deviation (sd_log) of ln(fAUC) were estimated from the observed data, and 5000 virtual observations were generated. For each simulated individual:

sim_fAUC/MIC = sim_fAUC/MIC

Target attainment was defined as:

target_attained = 1, if sim_fAUC/MIC ≥ target_threshold

target_attained = 0, if sim_fAUC/MIC < target_threshold

PTA was then calculated as:

PTA = P(fAUC/MIC ≥ target_threshold)

That is, the proportion of simulated individuals meeting the PK/PD target. PTA is widely used in antimicrobial dose optimization and translational PK/PD evaluation because it expresses target attainment at the population level rather than only through a single mean estimate [32].

### 2.11. Statistical Analysis

Raw drug concentration data underwent quality control (QC) prior to analysis. The QC process included checks for missing values, sample counts at each time point, and non-positive concentrations. Potential outliers were identified within each sample-timepoint stratum using the modified Z-score based on the median absolute deviation (MAD); this score was calculated on the log-transformed concentration scale when all concentration values were positive. Observations with an absolute modified Z-score > 3.5 were flagged as outliers and treated as missing values in the cleaned analysis dataset. No outlier assessment was performed when fewer than three evaluable observations were available, non-positive values were present, or the MAD equaled zero.

Pharmacokinetic parameters included the maximum concentration (Cmax) and the area under the concentration-time curve (AUC). The penetration rate (PR) was calculated as the ratio of free tissue concentration to plasma concentration (Ctissue/Cplasma). The distribution ratio of the antimicrobial from tissue to plasma (AUCtissue/AUCplasma) was determined by dividing the tissue AUC by the plasma AUC. Pharmacokinetic parameters derived from composite concentration-time profiles are presented as point estimates from the noncompartmental analysis. Variability was assessed by nonparametric bootstrapping (*n* = 2000), with results reported as bootstrap mean [95% confidence interval], whereas Tmax is reported as the bootstrap median [95% CI]. Statistical analyses were performed with R software (version 4.3.3). Statistical significance was set at *p* < 0.05.

## 3. Results

### 3.1. Reproductive System Contrast Imaging via the Novel Disposable Vas Deferens Injection Device

Using a novel disposable vas deferens injection device (Figure 1E), iodixanol was administered into the rat vas deferens, and reproductive system images were acquired with a micro-CT scanner (Figure 1D). The contrast images demonstrated preferential distribution along the reproductive tract after trans-vas-deferens delivery, which supported the feasibility of local administration.

### 3.2. NCA-Derived Plasma/Prostate Pharmacokinetic Parameters

Table 1 summarizes the plasma pharmacokinetic parameters derived by noncompartmental analysis (NCA) and the corresponding bootstrap summaries. The original NCA results showed that the IV group had substantially higher systemic exposure than the VS group. The composite plasma Cmax was 10.72 μg/mL for IV and 4.74 μg/mL for VS, while AUC0-24 was 28.59 and 15.47 μg·h/mL, respectively. Tmax occurred at 0.5 h for IV and 1.0 h for VS; these are observed sampling times rather than model-derived estimates. The bootstrap summaries were consistent with this pattern, with mean [95% CI] Cmax values of 10.72 [9.15, 12.35] μg/mL and 4.84 [2.28, 8.37] μg/mL, and AUC0-24 values of 28.64 [25.19, 32.28] and 15.53 [11.67, 19.91] μg·h/mL in the IV and VS groups, respectively.

Together, Table 1 shows that VS reduced systemic exposure relative to IV.

Table 2 presents the prostate pharmacokinetic parameters derived by NCA and their bootstrap summaries. In the original NCA analysis, Cmax was 52.02 μg/g in IV and 61.96 μg/g in VS, and AUC0-24 was 208.77 and 235.77 μg·h/g, respectively. Tmax occurred at 2 h for IV and 1 h for VS; these are observed sampling times rather than model-derived estimates. Bootstrap mean [95% CI] Cmax values were 51.98 [33.52, 65.98] μg/g for IV and 62.06 [39.64, 86.96] μg/g for VS, while AUC0-24 values were 208.44 [179.51, 235.93] and 236.37 [188.78, 285.96] μg·h/g. The tissue distribution factor fT was higher in VS than in IV (2.26 [1.58, 3.19] vs 1.06 [0.89, 1.30]), suggesting greater prostate selectivity despite similar absolute prostate exposure.

### 3.3. Concentration-Time Profiles and Time-Dependent Tissue Distribution of Levofloxacin

**Figure 2:**
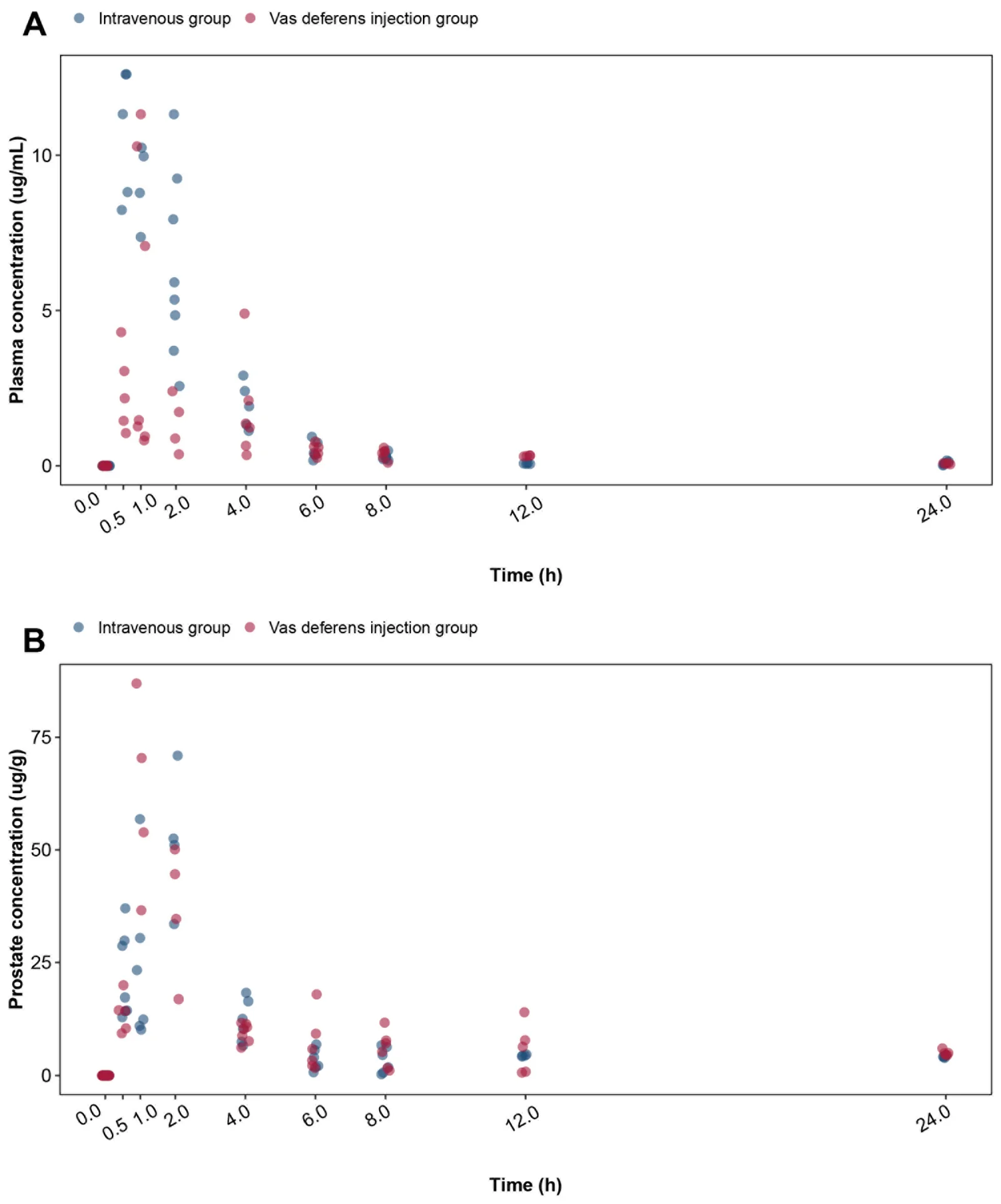
Plasma and prostate concentration-time profiles after intravenous injection and vas deferens injection. (**A**) Plasma concentration-time profiles in the intravenous injection (IV) group and the vas deferens injection (VS) group. (**B**) Prostate concentration-time profiles in the IV and VS groups. Individual observations are shown as points, solid lines indicate group means, and shaded ribbons represent mean ± standard deviation (SD).

Figure 2 illustrates the plasma and prostate concentration-time profiles for the IV and VS groups. In plasma, the IV group exhibited higher concentrations than the VS group during the early phase, whereas later time points showed a more gradual decline under VS. In the prostate, most time points did not show a clear between-group separation, but the VS group tended to retain higher concentrations at later times.

**Figure 3:**
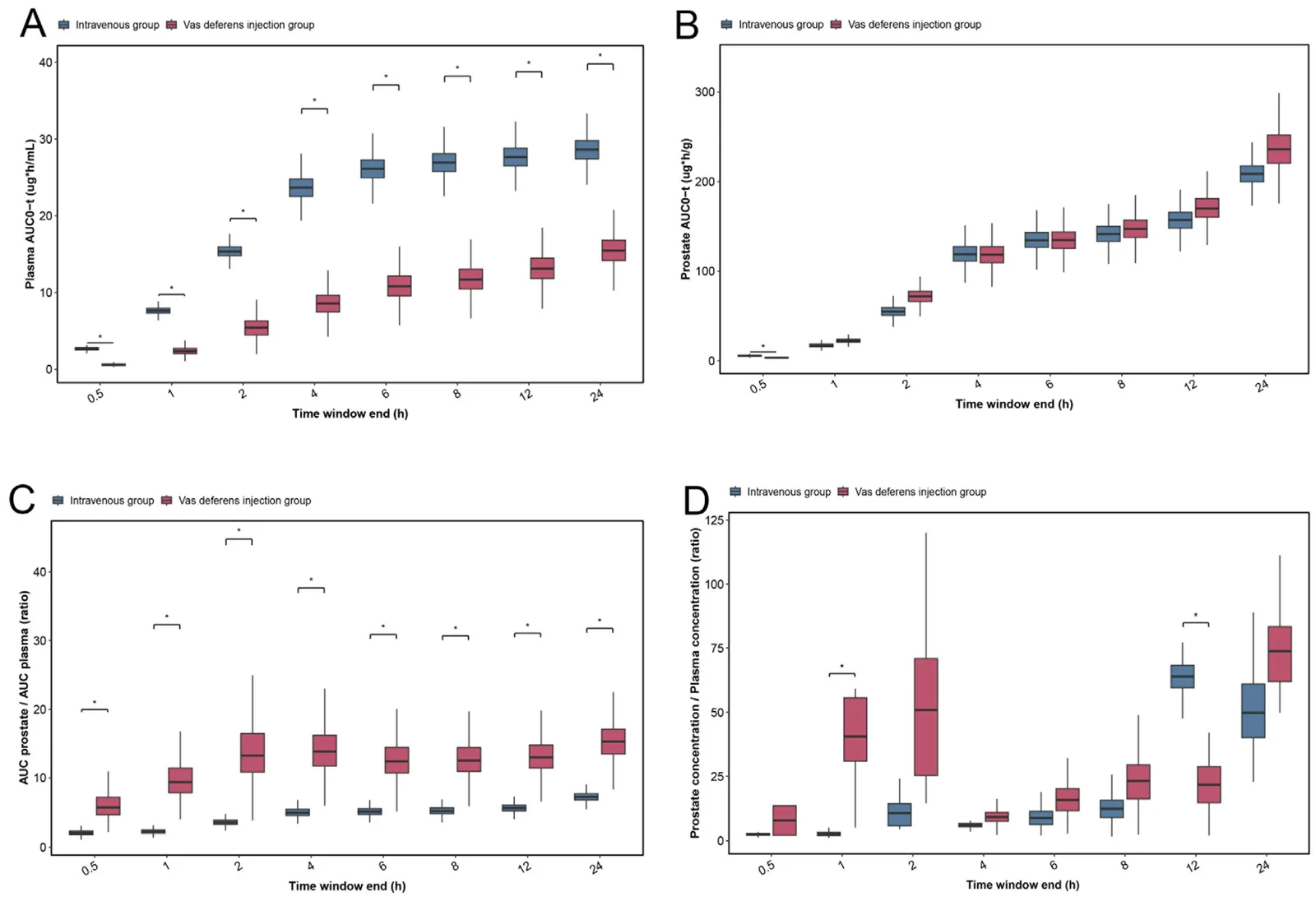
Time-dependent exposure and tissue distribution analyses based on partial area under the concentration-time curve and concentration ratios. (**A**) Bootstrap distributions of plasma partial area under the concentration-time curve from time 0 to t (AUC0-t) across predefined time windows in the IV and VS groups. (**B**) Bootstrap distributions of prostate partial AUC0-t across predefined time windows in the IV and VS groups. (**C**) Bootstrap distributions of the prostate-to-plasma partial AUC ratio across time windows. (**D**) Bootstrap distributions of the prostate-to-plasma concentration ratio at matched sampling times. * indicates *p* < 0.05 compared to the IV group.

Figure 3 further characterizes exposure differences in a time-dependent manner. Plasma partial AUC0-t values were significantly higher in the IV group.

In contrast, prostate partial AUC0-t values did not reach statistical significance across the assessed windows, although the VS group showed numerically higher exposure at the 24 h endpoint. When prostate exposure was normalized to plasma exposure, the prostate-to-plasma AUC ratio became significantly higher in the VS group. The prostate-to-plasma concentration ratio analysis showed a significant difference at 1 h and 12 h, but most other time points were not significant. Collectively, these findings indicate that VS reduced systemic exposure while increasing prostate-selective distribution. Because plasma and prostate concentrations at each scheduled time point were obtained from the same animal, this matched pointwise prostate-to-plasma ratio may help interpret the inter-animal variability of the VS route.

### 3.4. Bioequivalence-Style Relative Exposure Analysis

**Figure 4:**
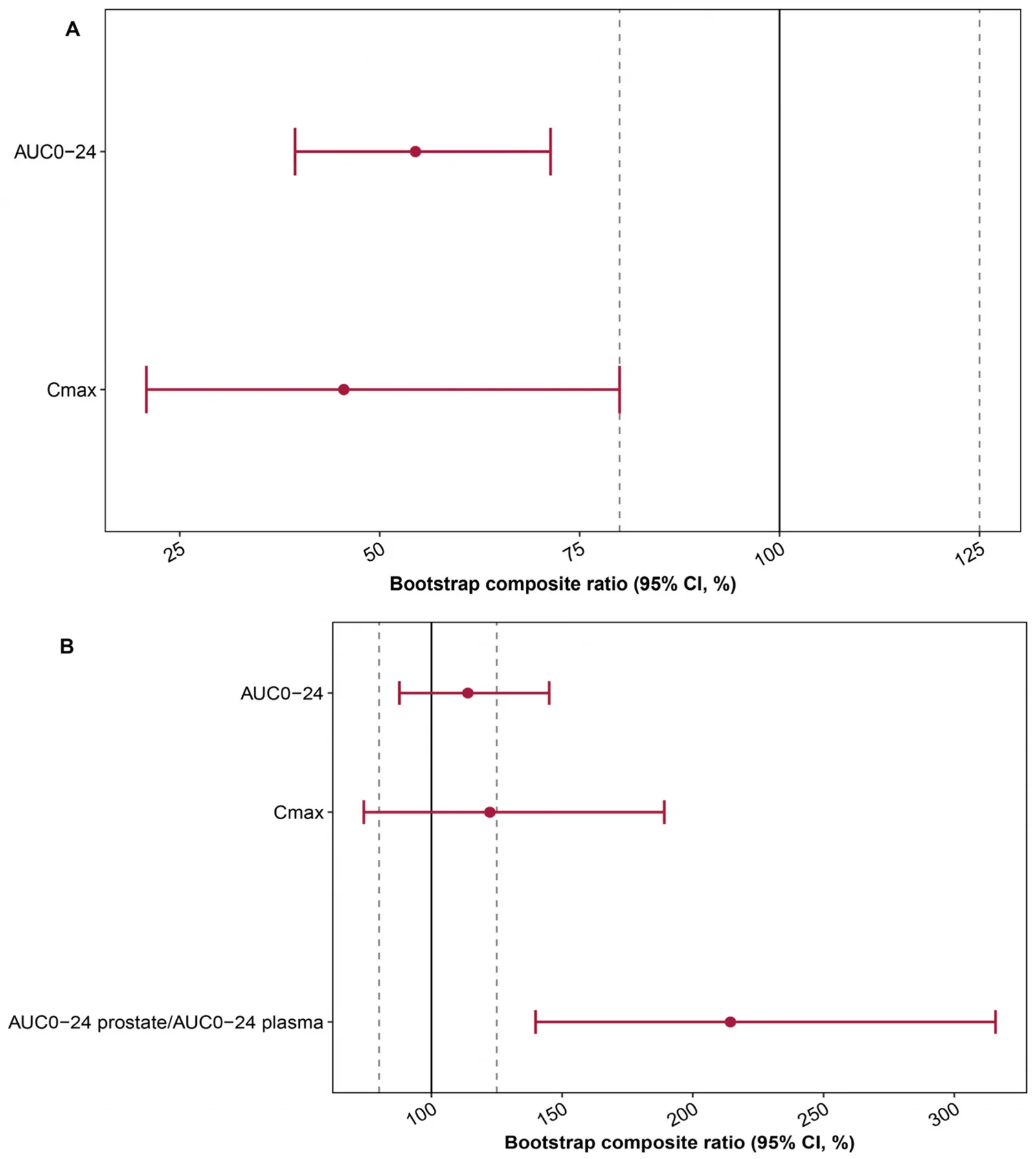
Relative exposure and bioequivalence-style comparison of pharmacokinetic parameters. (**A**) Forest plot of plasma pharmacokinetic parameters comparing the VS group with the IV group. (**B**) Forest plot of prostate pharmacokinetic parameters comparing the VS group with the IV group. Vertical reference lines indicate 80%, 100%, and 125%. Points indicate mean ratios and horizontal bars represent GMR and interval estimates.

In plasma (Figure 4A), the vas deferens injection group exhibited lower exposure than the intravenous group, with a bootstrap geometric mean ratio (GMR) of 54.1% (95% CI, 40.5–72.7%) for AUC0-24 and 43.3% (95% CI, 21.4–79.6%) for Cmax. The GMRs for AUC0-24 and Cmax were both well below 80%, indicating that VS substantially reduced systemic exposure relative to IV. In contrast, prostate exposure (Figure 4B) was generally maintained in the vas deferens injection group, with a bootstrap GMR of 112.6% (95% CI, 88.0–143.6%) for AUC0-24 and 117.9% (95% CI, 75.3–184.3%) for Cmax. The prostate-to-plasma AUC0-24 ratio was higher in the vas deferens injection group, with a bootstrap GMR of 208.4% (95% CI, 138.9–301.5%).

Taken together, Figure 4 shows that VS lowered plasma exposure while maintaining prostate exposure and substantially improving prostate selectivity relative to systemic exposure. This also demonstrates that this approach can result in a wider therapeutic window and less systemic burden.

### 3.5. Exploratory Levofloxacin Evaluation Against Prostate Infections

**Figure 5:**
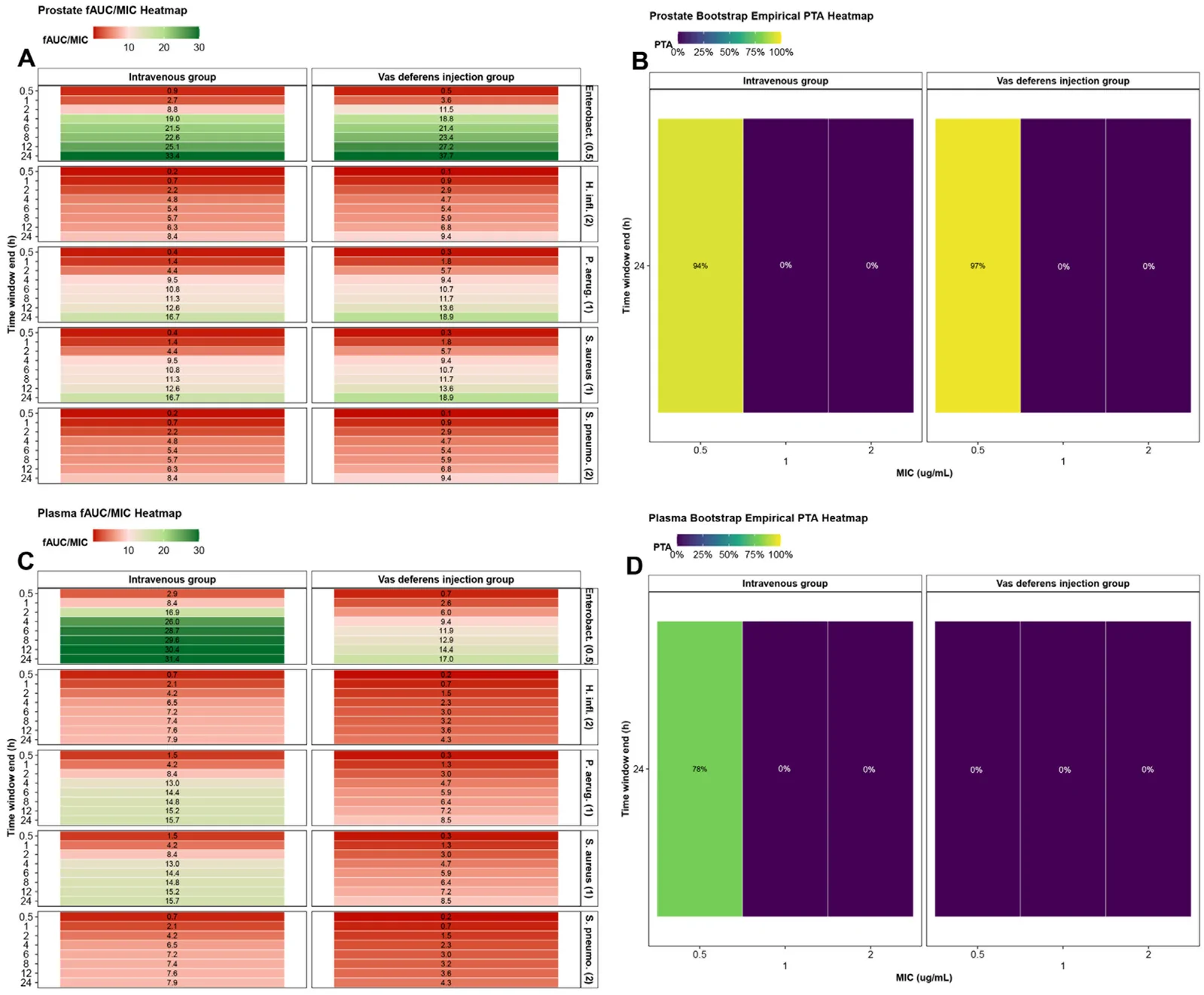
Pharmacodynamic evaluation in the target tissue based on free area under the concentration-time curve/minimum inhibitory concentration and probability of target attainment. (**A**) Bootstrap heatmap of prostate free area under the concentration-time curve/minimum inhibitory concentration (fAUC/MIC) values stratified by pathogen, representative minimum inhibitory concentration (MIC), time window, and treatment group. Tile color intensity indicates the magnitude of fAUC/MIC, and the overlaid values represent the corresponding estimates. MIC data are from WHO Pathogens Priority List Working Group. Discovery, research, and development of new antibiotics: The WHO priority list of antibiotic-resistant bacteria and tuberculosis. (**B**) Bootstrap heatmap of probability of target attainment (PTA) across selected MIC values and time windows in the prostate. Tile color intensity indicates PTA, and the overlaid values represent the estimated probabilities of reaching the predefined pharmacokinetic/pharmacodynamic target. (**C**) Bootstrap heatmap of plasma-free area under the concentration-time curve/minimum inhibitory concentration (fAUC/MIC) values stratified by pathogen, representative minimum inhibitory concentration (MIC), time window, and treatment group. (**D**) Bootstrap heatmap of probability of target attainment (PTA) across selected MIC values and time windows in the plasma. Tile color intensity indicates PTA, and the overlaid values represent the estimated probabilities of reaching the predefined pharmacokinetic/pharmacodynamic target.

In the prostate at the 24 h time window, for Enterobacteriaceae with MIC = 0.5 μg/mL, the fAUC/MIC values were approximately 33.4 in the IV group and 37.7 in the VS group in Figure 5A. By contrast, in the plasma at the 12 h and 24 h time windows, for Enterobacteriaceae, the fAUC/MIC value of the intravenous group was substantially higher than that of the vas deferens injection group in Figure 5C.

The Monte Carlo-derived PTA analysis supported this conclusion in Figure 5B. In the prostate at the 24 h window, when MIC = 0.5 μg/mL, the PTA values were 94% for IV and 97% for VS. In the plasma at the 24 h window, when MIC = 0.5 μg/mL, the PTA value of the intravenous group was substantially higher than that of the vas deferens injection group in Figure 5D.

These findings also suggest that a drug with vas deferens injection can result in a wider therapeutic window and less systemic burden.

## 4. Discussion

The present study shows that trans-vas-deferens administration altered the exposure pattern of levofloxacin in a direction that was favorable for local prostate delivery. Across the concentration-time profiles, partial AUC analyses, and the bioequivalence-style comparison, the VS regimen consistently produced lower plasma exposure but maintained or numerically increased prostate exposure. The clearest separation between regimens was observed for systemic metrics, where plasma AUC0-24 and Cmax were both reduced under VS, whereas the prostate tissue distribution factor fT increased markedly. This combination supports the central pharmacokinetic premise of local delivery, namely that the route can reduce systemic burden while preserving drug availability at the target site. The trans-vas-deferens procedure may also be associated with procedure-related risks, such as mechanical trauma related to puncture or catheterization, injectate reflux or leakage, and local complications including pain and scrotal swelling. These considerations should be taken into account when interpreting the potential translational value of this method.

These findings are consistent with the broader rationale for localized drug delivery in prostate disease. Intraprostatic injection, rectal delivery, ultrasound-assisted delivery, and nanocarrier-based systems have all been explored to increase local exposure while limiting off-target distribution [16,17,19,20,21,22,33,34,35,36,37]. However, many localized approaches either require imaging guidance, involve more invasive manipulation of the gland, or remain technically complex for routine translation. The present vas-deferens route is conceptually distinct in that it uses the natural continuity of the male reproductive tract to reach the prostate and therefore may offer a practical alternative for targeted administration.

The time-dependent analyses help explain why the VS regimen yielded a higher degree of prostate selectivity. IV produced greater early plasma exposure, whereas VS showed a more gradual decline in plasma and a later exposure advantage in the prostate. In parallel, the prostate-to-plasma AUC ratio became significantly higher in the VS group during the middle and late phases, and the pointwise concentration ratio showed the same direction at 12 h. The Figure 4 forest plots extend this interpretation: in plasma, the GMRs for AUC0-24 and Cmax shifted toward reduced exposure under VS, whereas in the prostate, the corresponding parameters together with AUC0-24 prostate/AUC0-24 plasma were centered around preservation or increase in local exposure. Together, these results indicate that the principal advantage of VS was not simply a higher single peak concentration, but a more favorable balance between local retention and systemic exposure.

The observed distribution pattern is also biologically plausible. Drug movement after intravasal administration likely depends on anatomic continuity among the vas deferens, seminal vesicles, ejaculatory ducts, and the prostate, with subsequent diffusion into peri-prostatic tissues [15,38]. This pathway differs from standard systemic administration, which must overcome the blood-prostate barrier before meaningful tissue exposure can be achieved [7,15,24,25]. Studies in other organs have shown that levofloxacin distribution varies substantially according to barrier function, with relatively high exposure in tissues such as the lung and pancreas and lower exposure in more protected organs such as the brain [39,40,41,42,43,44]. Previous work has also suggested that prostate penetration may change with developmental stage and inflammatory status, indicating that the blood-prostate barrier is dynamic rather than fixed [45]. Against that background, the enhanced prostate selectivity seen with VS is pharmacologically credible.

The exploratory PK/PD evaluation provides additional context for the translational relevance of this delivery route. In the prostate at 24 h, VS yielded a numerically higher fAUC/MIC (37.7 vs. 33.4 for Enterobacteriaceae at an MIC of 0.5 μg/mL) and a correspondingly higher PTA (97% vs. 94%) compared with IV. In the plasma, both fAUC/MIC and PTA were consistently lower in the VS group across the assessed time windows, consistent with the reduced systemic exposure observed in the pharmacokinetic analysis. Because fluoroquinolone activity is primarily driven by free-drug exposure relative to MIC, this pattern suggests that vas deferens administration may improve local antibacterial target attainment while reducing systemic drug burden. However, these PK/PD indices were estimated using literature-derived free fractions rather than directly measured unbound concentrations, and formal statistical comparisons between regimens were not performed; the results should therefore be interpreted as exploratory [28,29,30,31].

In this study, the prostate pharmacokinetic parameters (Cmax and AUC0-24) in the VS group were numerically higher than those in the IV group, although bootstrap confidence intervals showed considerable overlap between regimens (Table 2). As shown in Figure 1D, contrast agent administered via the vas deferens was distributed along the reproductive tract. This distribution pattern suggests that a variable fraction of the administered dose may be lost via urethral reflux or washout during urination, which could contribute to inter-individual variability in early drug concentrations. Outliers were identified and excluded using a prespecified statistical criterion. The matched prostate-to-plasma concentration ratio obtained within the same animal at each time point may help explain part of the observed variability.

Several limitations should be acknowledged. First, this was a rat pharmacokinetic study rather than a therapeutic efficacy study in an infected prostatitis model, so the PK/PD results should be interpreted as translational indicators rather than direct proof of clinical cure. Second, the sample size was limited and some prostate confidence intervals remained wide, especially for Cmax and fT. Third, free-drug exposure was estimated from literature-based free fractions rather than measured directly in the present experiment. Fourth, contrast imaging suggested partial distribution of the injectate beyond the prostate (including the bladder and urethra), indicating that variable drug loss via retrograde flow or urinary washout may have contributed to inter-individual variability and attenuated the observed prostate exposure in the VS group.

Future work should include infected models, direct free-drug sampling where feasible, comparative dose-ranging studies, optimizing the vas deferens injection strategy (to minimize drug loss and enhance retention within the reproductive system), and ultimately clinical validation in patients with prostate disease.

## 5. Conclusions

Trans-vas-deferens administration reduced systemic levofloxacin exposure while maintaining or enhancing prostate exposure, thereby improving prostate-selective drug distribution. The associated increase in prostate fAUC/MIC and PTA supports further evaluation of this route as a targeted treatment strategy for prostate diseases. This potential advantage should be balanced against the procedure-related risks of trans-vas-deferens administration, including mechanical injury from puncture/catheterization, injectate reflux or leakage, and local adverse reactions such as pain and scrotal swelling.

## Figures and Tables

**Table 1:** PK parameters determined by NCA of the plasma profiles.

Parameters	Plasma ^a^		Plasma (Bootstrap = 2000) ^b^	
	IV	VS	IV	VS
Cmax, μg/mL	10.72	4.74	10.72 [9.15, 12.35]	4.84 [2.28, 8.37]
Tmax, h	0.5	1	0.50 [0.50, 1.00]	1.00 [0.50, 1.00]
AUC0_24, μg·h/mL	28.59	15.47	28.64 [25.19, 32.28]	15.53 [11.67, 19.91]

Notes: Cmax, maximum observed composite concentration; Tmax, time of observed composite Cmax; AUC0_24, AUC from zero to 24 h; IV, intravenous injection; VS, vas deferens injection; ^a^, each point represents a single animal sampled at a terminal time point; ^b^, Tmax is reported as the bootstrap median [95% CI], whereas the remaining parameters are reported as the bootstrap mean [95% CI].

**Table 2:** PK parameters determined by NCA of the prostate profiles.

Parameters	Prostate ^a^		Prostate (Bootstrap = 2000) ^b^	
	IV	VS	IV	VS
Cmax, μg/g	52.02	61.96	51.98 [33.52, 65.98]	62.06 [39.64, 86.96]
Tmax, h	2	1	2.00 [2.00, 2.00]	1.00 [0.50, 2.00]
AUC0_24, μg·h/g	208.77	235.77	208.44 [179.51, 235.93]	236.37 [188.78, 285.96]
fT	1.06	2.22	1.06 [0.89, 1.30]	2.26 [1.58, 3.19]

Notes: Cmax, maximum observed composite concentration; Tmax, time of observed composite Cmax; AUC0_24, AUC from zero to 24 h; IV, intravenous injection; VS, vas deferens injection; The tissue distribution factor (fT) was calculated as [AUCtissue,free/(fuplasma × AUCplasma,total)], where fut_plasma = 0.55. ^a^, each point represents a single animal sampled at a terminal time point; ^b^, Tmax is reported as the bootstrap median [95% CI], whereas the remaining parameters are reported as the bootstrap mean [95% CI].

## Data Availability

Data will be made available upon request.

## Supplementary Materials

The following supporting information can be downloaded at: https://www.mdpi.com/article/10.3390/pharmaceutics18080931/s1, Figure S1: Representative calibration curves of levofloxacin in rat plasma (upper panel) and prostate tissue (lower panel). Linear regression showed good linearity over the tested concentration range, with y = 6.7213x + 0.0720 (R^2^ = 0.9998) for plasma and y = 5.0565x − 0.0903 (R^2^ = 0.9957) for prostate tissue.
