# Supplementary material for: Targeted Prostatic Delivery of Levofloxacin via a Novel Vas-Deferens Injection Device: A Pharmacokinetic Study in Rats
Source: Pharmaceutics. 2026 Jul 29;18(8):931. doi: 10.3390/pharmaceutics18080931 (PMC13516220; doi:10.3390/pharmaceutics18080931)
Supplement: Supplementary file 1 [file pharmaceutics-18-00931-s001.zip › pharmaceutics-4453977-supplementary.pdf]

# Supplementary Materials: Targeted Prostatic Delivery of Levofloxacin via a Novel Vas-Deferens Injection Device: A Pharmacokinetic Study in Rats

**Haiming Cao, Junjie Wu, Mingwei Zhan, Bo Ma, Qi Zhang and Xuejun Shang**

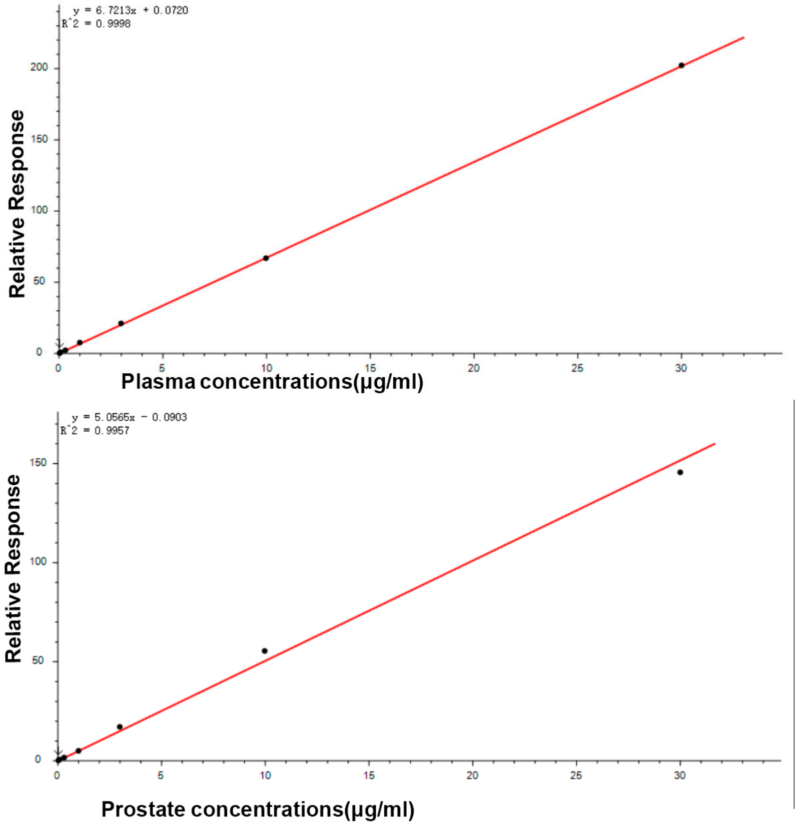


**Figure S1.** Representative calibration curves of levofloxacin in rat plasma (upper panel) and prostate tissue (lower panel). Linear regression showed good linearity over the tested concentration range, with y = 6.7213x + 0.0720 (R^2 = 0.9998) for plasma and y = 5.0565x − 0.0903 (R^2 = 0.9957) for prostate tissue.
